# Electrochemical SEIRAS Analysis of Imidazole-Ring-Functionalized Self-Assembled Monolayers

**DOI:** 10.3390/ma15207221

**Published:** 2022-10-17

**Authors:** Vaidas Pudžaitis, Martynas Talaikis, Rita Sadzevičienė, Linas Labanauskas, Gediminas Niaura

**Affiliations:** 1 Department of Organic Chemistry, Center for Physical Sciences and Technology (FTMC), Sauletekio Ave. 3, LT-10257 Vilnius, Lithuania; 2 Department of Bioelectrochemistry and Biospectroscopy, Institute of Biochemistry, Life Sciences Center, Vilnius University, Sauletekio Ave. 7, LT-10257 Vilnius, Lithuania

**Keywords:** SEIRAS, histidine, imidazole, electrochemistry, DFT, hydrogen-bonding interaction

## Abstract

An essential amino acid, histidine, has a vital role in the secondary structure and catalytic activity of proteins because of the diverse interactions its side chain imidazole (Im) ring can take part in. Among these interactions, hydrogen donating and accepting bonding are often found to operate at the charged interfaces. However, despite the great biological significance, hydrogen-bond interactions are difficult to investigate at electrochemical interfaces due to the lack of appropriate experimental methods. Here, we present a surface-enhanced infrared absorption spectroscopy (SEIRAS) and density functional theory (DFT) study addressing this issue. To probe the hydrogen-bond interactions of the Im at the electrified organic layer/water interface, we constructed Au-adsorbed self-assembled monolayers (SAMs) that are functionalized with the Im group. As the prerequisite for spectroelectrochemical investigations, we first analyzed the formation of the monolayer and the relationship between the chemical composition of SAM and its structure. Infrared absorption markers that are sensitive to hydrogen-bonding interactions were identified. We found that negative electrode polarization effectively reduced hydrogen-bonding strength at the Im ring at the organic layer–water interface. The possible mechanism governing such a decrease in hydrogen-bonding interaction strength is discussed.

## 1. Introduction

**Figure 1:**
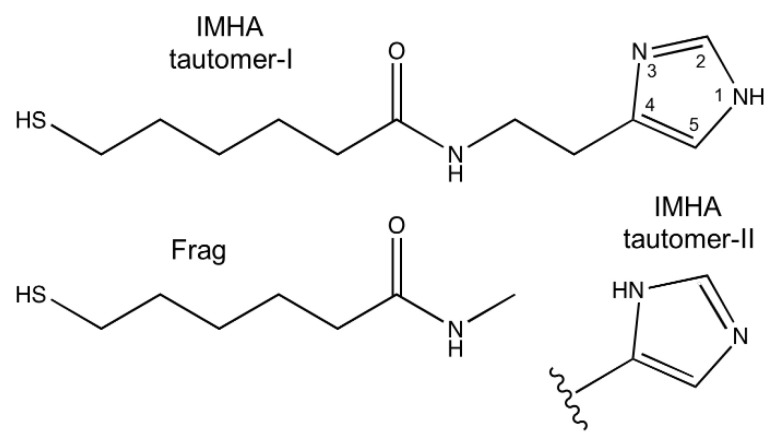
Chemical structures of N-(2-(1H-imidazol-4-yl)ethyl)-6-mercaptohexanamide (IMHA) and 6-mercapto-N-methylhexanamide (molecular fragment, Frag). Atom numbering indicated for imidazole ring. Two tautomeric forms of imidazole are shown.

Histidine involvement in protein architecture and catalytic activity is primarily determined by the diverse range of interactions that its side chain imidazole (Im) can take part in [1]. Im acts as an aromatic system, participates in hydrogen-bond (H-bond) interactions, and coordinates metal (II) cations through the nitrogen’s lone electron pair [2]. Im has a pKa value of ~5.9; therefore, in physiological conditions, only one nitrogen is protonated, which results in two possible tautomeric forms. Tautomer-I (T-I) is protonated at N1 atom (N1–H, N3); Tautomer-II (T-II) is protonated at N3 (N1, N3–H). Tautomeric forms of imidazole ring are shown in Figure 1. Because of the slightly more energetically favorable form, T-I dominates neutral pH solutions at room temperature [3]. The tautomeric equilibrium can change depending on the chemical Im group environment, temperature, intermolecular interactions, and other external conditions. For example, a clear T-I to T-II shift in imidazole-containing compounds was found to be induced by the chemisorption on a metal surface [4].

Hydrogen bonding is among the most important interactions that govern protein folding, molecular conformation, and packing. Notable is the Im ring, which in neutral form can participate in both hydrogen accepting and donating interactions. However, studying these H-interactions at the electrified interface is a challenging task due to the lack of appropriate experimental techniques. Vibrational spectroscopy is highly sensitive to the local environment and structure of probed molecules, intermolecular forces, and electric fields. In particular, Raman spectroscopy provides detailed information on the Im structure and the interactions with divalent metal cations, both in natural biomolecules and model compounds [3,4,5,6,7,8,9,10,11,12]. Fourier-transform infrared absorption spectroscopy (FTIR) exceeds Raman by a many-orders-of-magnitude-larger process cross section, which is especially beneficial in dynamic studies and in the cases of a low number of molecules under investigation.

Surface-enhanced infrared absorption spectroscopy (SEIRAS), pioneered by Osawa [13], has become a valuable tool in the analysis of Au or Ag surface-adsorbed supramolecular systems in situ [14]. In recent years, the method became technologically simpler, which has even led to its commercialization [15,16]. Various techniques of thin Au-layer preparation were devised, such as electroless, vapor, and electrochemical deposition [17], and also hybrid ones [18], which offer increased signal-to-noise ratio, Au-film rigidness, and even enable multiple reuses of the same film. The most readily available method today remains electroless deposition since it is relatively fast and requires less investment in equipment. With the right amount of care taken due to harmful chemicals used, the method is not complicated and is even time-efficient [19].

In this study, we used an electrochemical-SEIRAS method to attain a deeper understanding of the Im ring’s interactions with the local environment at the organic layer–water interface on an electrically polarized electrode. The Im-ring-functionalized self-assembled monolayers were formed on the Au surface from the N-(2-(1H-imidazol-4-yl)ethyl)-6-mercaptohexanamide (IMHA) compound. The SEIRAS technique provided insights into the adsorption and reorientation dynamics of the SAM molecules with an emphasis on the changes in the functional Im ring group. Spectroscopic markers for the H-bond interactions at the Im group were identified and the analysis of their alterations at the electrified interface was provided. Spectroscopic findings were backed by quantum chemistry calculations, which are prominent when studying hydrogen-bonding interactions [20]. Our findings will shed some light on the interfacial behavior of the amino acid, histidine, which has an essential role in protein structure and activity and will be beneficial in surface chemistry applications.

## 2. Materials and Methods

### 2.1. Materials

Acetone (99.9%), sodium acetate (98.5%), H_2_O_2_ (30%), Na_2_SO_3_ (98%), NH_4_Cl (99.5%), NH_4_F (98%), HF (48%), Na_2_S_2_O_3_·5H_2_O (ACS grade), NaAuCl_4_·2H_2_O (99%), and 4-imidazole-methanol (97%) were purchased from the chemical suppliers Sigma-Aldrich (St. Louis, MO, USA), Fluka (Buchs, Switzerland), and Merck (Darmstadt, Germany); ethanol-d_6_ (99 atom% D) and methanol-d_4_ (99 atom% D) were from Roth (Karlsruhe, Germany). Water-based diamond particle suspensions (3, 1, and 0.25 µm) were bought from QATM (Mammelzen, Germany). Deionized water (18.2 MΩ·cm) from Direct-Q 3UV purification system (Merck, Germany) was used to prepare solutions. Au-plating solutions were deaerated with nitrogen gas before use. Silicon crystal (face-angled crystal, FAC) was bought from Pike Technologies (Fitchburg, WI, USA). N-(2-(1H-imidazol-4-yl)ethyl)-6-mercaptohexanamide (IMHA) and 6-mercapto-N-methylhexanamide (molecular fragment, Frag) were synthesized in-house. The synthesis details are published elsewhere [21]. IMHA and Frag hydrogen atoms at N and S were substituted with deuterium by dissolving the compounds in ethanol-d_6_ and then evaporating the solvent in a heated ultrasonic bath.

### 2.2. Electroless Au Plating

**Figure 2:**
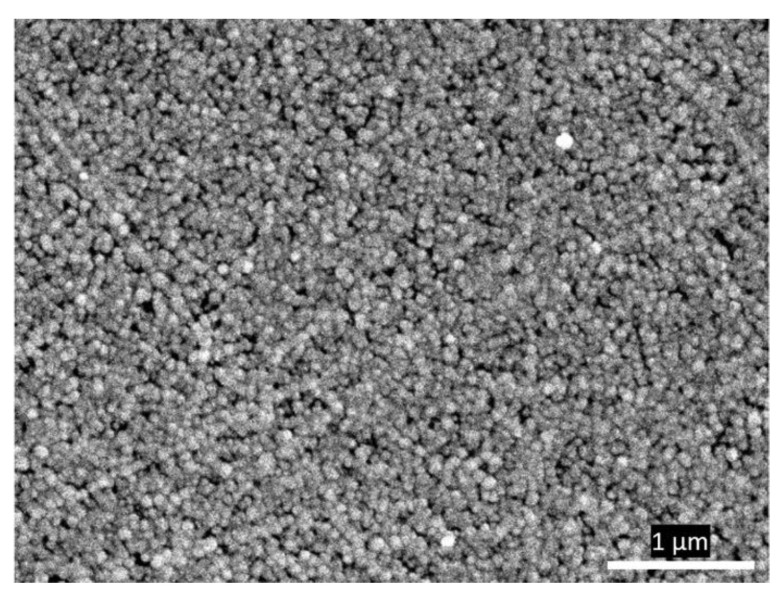
SEM image of the gold film deposited on a silicon prism. Magnification was ×25,000.

The reflecting plane of the Si crystal was polished by hand using a polishing pad (QATM, Mammelzen, Germany) and water-based diamond suspensions starting with 3 µm (5 min), then 1 µm (5 min), and 0.25 µm (20 min). Polishing was performed by shaping the “∞” symbol and turning the crystal by 90 degrees every 15 s, with thorough water rinsing in between each polishing step. Then, the mirror-polished Si crystal was ultrasonicated with acetone and water twice, for 10 min each time, and dried under a nitrogen stream. The prism sides were protected with 0.1 mm Teflon ribbon to prevent coatings from damage. Fresh Au-plating mixture was prepared in a Teflon container and stirred continuously under a nitrogen blanket. For that, equal volumes of (1) salt solution (0.15 M Na_2_SO_3_, 0.05 M Na_2_S_2_O_3_, 0.05 M NH_4_Cl), (2) NH_4_F (20 wt%), (3) HF (2 wt%), and (4) NaAuCl_4_ (0.03 M) were mixed to at least 2 mL in total. The (4) solution was freshly prepared for every plating. To hydrogenate the Si surface, the polished crystal was incubated with 1 mL HF (2 wt%) for 3 min, after which it was removed and 1 mL Au-plating mixture was added for another 3–4 min in room-temperature conditions. During Au plating, the crystal surface attained a bright yellow color. Reaction was stopped by transferring the crystal directly into a water bath for 15 min. SEM image in Figure 2 shows the formation of homogeneous nanostructures of approximately 50 nm in diameter. 

### 2.3. Attenuated Total Reflection (ATR)-SEIRAS Setup and Au-Film Activation

Spectral measurements were carried out by using Vertex 80v spectrometer (Bruker, Ettlingen, Germany) equipped with a liquid-nitrogen-cooled narrow-band MCT (HgCdTe) detector working at a 40 kHz scanner speed. The resolution was set to 4 cm^−1^ and aperture to 2 mm; sample and background scans were collected from 50 and 100 iterations, accordingly. Freshly prepared nitrogen-blow-dried Au/Si crystal was assembled into a VeeMax III variable angle accessory with Jackfish cell J1F (Pike Technologies, Fitchburg, WI, USA). The angle of the ATR unit was set to 63 degrees. After assembly, the spectrometer was purged with dry air overnight to eliminate residual water vapor from the spectrometer chambers. 

Gold layer of the Si crystal was electropolished and additionally activated in pH 5.8 sodium acetate solution (0.1 M) by performing cyclic voltammetry (CV) scans, controlled by PGSTAT101 potentiostat (Methrom, Riverview, FL, USA). A three-electrode system comprised the gold layer as working electrodes, reference Ag/AgCl, and counter Pt electrodes. A cell filled with sodium acetate solution (0.1 M) was purged with nitrogen for 30 min and CV was performed, starting in the range of ±200 mV from open circuit potential (OCP) at 20 mV/s speed. After 3 full cycles, potential range was increased by 100 mV towards the anodic direction. The procedure continued with 3 time repeats for each 100 mV increment until gold oxidation became detectable. SEIRAS measurements at designated CV steps were completed to confirm surface cleaning. Reference (sample) spectra were collected at –100 mV (600 mV) to identify the marker band of the gold surface-adsorbed acetate. The υν(COO^–^) mode near 1400 cm^−1^ gained intensity with increasing surface cleanness at later steps of the CV treatment. The couple of milli-absorption intensities of the ν(COO^–^) mode is sought after since longer CV treatment increases the risk of gold-layer delamination.

### 2.4. Self-Assembled Monolayer (SAM) Formation

Gold layer was washed several times with H_2_O before collecting the reference SEIRAS spectrum for later use. Then, the cell was thoroughly rinsed with ethanol without letting the gold surface to dry. Approximately 0.2 mL of IMHA, Frag, or IMHA/Frag ethanol mixture (Figure 1) was injected into a cell to a total 0.5 mM concentration and incubated for 90 min. Monolayers were formed from 100, 50, 20, and 0 mol% IMHA solutions and denoted as IMHA, IMHA/Frag (1/1), IMHA/Frag (1/5), and Frag. Each experiment was repeated in the same order by using the deuterated solvents (D_2_O and ethanol-d_6_).

### 2.5. Attenuated Total Reflection Spectroscopy (ATR-FTIR) Measurements

ATR-FTIR spectra of IMHA and its fragment were measured using Alpha spectrometer (Bruker, Ettlingen, Germany) equipped with a diamond ATR accessory and DTGS detector working at a 7.5 kHz scan speed. The spectral resolution was set to 4 cm^−1^ and 200 scans were co-added for sample and background channels. 

### 2.6. Scanning Electron Microscopy (SEM)

The SEM images were acquired by JSM-IT200 InTouchScope^TM^ (Jeol, Kyoto, Japan) microscope working at 20 kV accelerating voltage and a secondary electron detector. 

### 2.7. Density Functional Theory (DFT) Modeling

Theoretical modeling was performed using Gaussian 09W [22]. Geometry optimization and frequency calculations were completed by using the B3LYP-functional and the 6-311++G(2d,p)-basis sets without anharmonic correction. Calculated frequencies and intensities were scaled according to the procedure described elsewhere [23]. In short, wavenumber axis was scaled according to $\nu ’=\left(1-\left(1-\alpha^{F}\right)\cdot \frac{\nu -\nu^{0}}{\nu^{F}-\nu^{0}}\right)\cdot \nu$, where ν and ν’ are original and corrected wavenumbers, respectively. Parameters were α^F^ = 0.97, ν^F^ = 4000, and ν^0^ = 600. No imaginary wavenumbers were obtained in the calculated spectra.

## 3. Results and Discussion

### 3.1. Assignment of Spectral Bands of IMHA and Molecular Fragment

**Figure 3:**
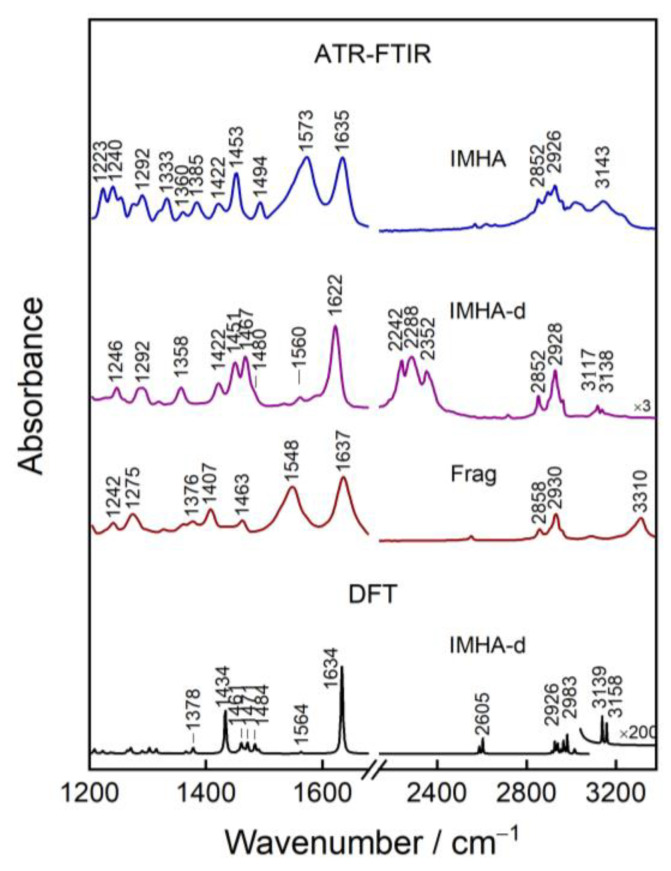
ATR-FTIR and DFT spectra of bulk IMHA and molecular fragment. IMHA-d refers to S–D- and N–D-substituted IMHA molecules.

The IMHA comprises four structure elements: (1) the thiol group (–SH) that interacts with Au surface, (2) the alkane chain ((–CH_2_–)_5_) spacer, (3) the amide group that increases SAM stability [24], and (4) the functional Im ring. In order to have control over the imidazole ring concentration at the interface, a surface backfiller (molecular fragment, Frag) was introduced into the monolayers. Molecular fragment has a similar chemical structure to IMHA, except for the Im ring, which is substituted with the methyl group (Figure 1). Figure 3 shows ATR-FTIR spectra of IMHA, Frag, and an IMHA molecule recrystallized from ethanol-d_6_ (IMHA-d). Assignments of the Im ring and amide spectral modes, based on the literature and our density functional theory (DFT) calculations, are given in Table 1 [4,6,7,8,11,25,26,27,28,29].

The two most pronounced bands in the ATR-FTIR spectrum of solid IMHA are assigned to the Amide-I (Am-I) vibration at 1635 cm^−1^, which is mainly C=O coupled with the out-of-phase C–N stretching and C–C–N bending motion, and Amide-II (Am-II) at 1573 cm^−1^, which is associated with the NH bending coupled with the C–N stretching [30,31]. Due to recrystallization in ethanol-d_6_, the mobile N-hydrogens exchange with D from the solvent (H/D exchange), which decreases the vibrational frequencies of the Am-I and Am-II modes to 1622 and 1467 cm^−1^, respectively. Deuterium substitution also breaks the vibrational coupling between ND bending and C–N stretching and effectively changes the direction of the transition dipole moment (TDM) vector of the Am-II vibration [32]. The aliphatic chains of IMHA and Frag give rise to modes near 2852 and 2926 cm^−1^ ascribed to symmetric and asymmetric methylene-stretching motion and also scissoring deformation of CH_2_ at 1451–1463 cm^−1^. We identify symmetric deformation of the terminal methyl group, δ_s_(CH_3_), of the molecular fragment for which the frequency at 1407 cm^−1^ is affected by the induction effect from the neighboring nitrogen of the amide group [33]. The complex N–H stretching pattern above 3000 cm^−1^ upon H/D exchange shifts to 2240–2350 cm^−1^ region, where three well-defined bands emerge. Medium intensity features at 2352 and 2288 cm^−1^ are linked with Amide-A’ and Amide-B’ [34,35]. The 2242 cm^−1^ mode in the IMHA spectrum is assigned to the vibration of the imidazole ring. The H/D exchange allowed for the identification of weak imidazole ring features otherwise obscured by strong infrared absorption bands. Modes at 3138 and 3117 cm^−1^ in the IMHA-d spectrum are associated with ν(C5–H) and ν(C2–H) vibrations, respectively—2242 cm^−1^ with N1–D stretching and 1560 cm^−1^ with ν(C4=C5). In the IMHA spectrum, the mode at 1494 cm^−1^ is easily recognized as C2–N3 stretch coupled with in-plane C2H bending of the Im ring, which in the IMHA-d spectrum appears as a shoulder at 1480 cm^−1^ (1484 cm^−1^ in DFT for IMHA) [27,29].

### 3.2. SEIRAS Analysis of the Formation of Self-Assembled Monolayer

**Figure 4:**
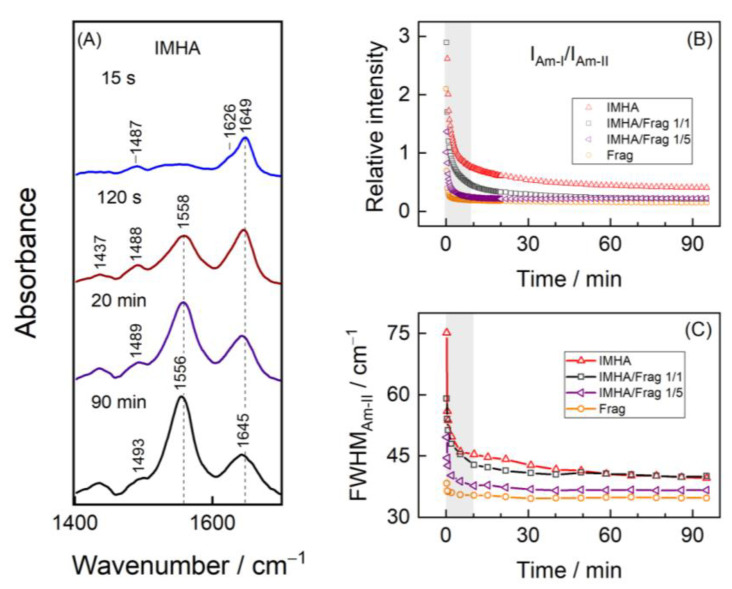
(**A**) SEIRAS spectra of IMHA SAM in ethanol adsorption solution at different incubation times. (**B**) The temporal evolution of the Am-I/Am-II intensity ratio of SAMs composed of IMHA, Frag, and their mixtures (1/1 and 1/5). (**C**) FWHM of Am-II spectral mode of different composition SAMs.

Figure 4A shows the time-dependent adsorption SEIRAS spectra of the IMHA monolayer formation in the ethanol incubation solution. The planar amide bond, –CO–NH–, gives rise to Am-I and Am-II vibrations, for which the transition dipole moments (TDM) are orientated perpendicularly to each other (Figure S1) [25]. Metal surface selection rule states that the intensities of spectral modes scale with respect to the TDM vector projection to the surface normal [36]; therefore, any changes in the amide plane orientation will be reflected in the altered Am-I and Am-II intensity patterns, which, conveniently, could be expressed as the intensity ratio Am-I/Am-II. After 15 s of incubation, the SEIRAS spectrum already exhibited clear features of Am-I (1626 cm^−1^ and shoulder at 1649 cm^−1^) and C–N stretching of the imidazole ring (1487 cm^−1^). At increasingly longer times, the Am-II mode at 1558 cm^−1^ emerged and subsequently became the predominant band. In previous studies, the Am-II was found to be the most intense infrared absorption mode for neat SAMs on atomically smooth Au surfaces formed from alkanethiol molecules with the intrachain amide group [37,38,39,40]. Strong Am-II is related to the amide C–N bond oriented perpendicular to the surface.

The wavenumbers of the amide spectral modes are telltale of the H-bond strength. The Am-I is predominantly sensitive to the H-bonding state of the amide’s carbonyl group, whereas the Am-II mode is mostly sensitive to the hydrogen-bonding interactions at the N–H site [41]. The weakening of the H-bonding interactions at C=O···H results in upshifted Am-I frequencies, while the weakening at the N–H···O results in downshifted Am-II frequencies [25,40,42]. Based on the changes in the wavenumbers of amide modes, we deduce that the formation of SAM adversely affects the intrachain packing of IMHA molecules at the amide group. The bulk IMHA molecules in the ATR-FTIR spectrum exhibited an Am-II absorption maximum at 1573 cm^−1^, which, upon adsorption on the Au surface, shifted to 1552 cm^−1^ (at 15 s). In the next five minutes, frequency upshifted to 1559 cm^−1^ but then slowly downshifted by several cm^−1^ in a time course of 90 min (Figure S2). Such a difference between the ATR-FTIR and SEIRAS modes (δ = 14–21 cm^−1^) shows a drastic reduction in H-bonding strength. Am-I, on the other hand, showed only a small-scale steady frequency decrease in the first five minutes, after which the changes remained minimal. Figure 4B clearly shows that, within the first 10 min of adsorption, the Am-I/Am-II ratio sharply decreased from 5.2 to 0.8 for the IMHA SAM, meaning that reorientation into more vertically aligned amide planes for molecules occurs concurrently with the adsorption on the Au surface. Such an alignment is necessary for the rigid lateral H-bond network formation of molecules in the monolayer.

To allow control over the IMHA molecule concentration on the surface and to enhance the motional flexibility of the Im group, we have synthesized and introduced molecular fragments into the monolayer composition. The increasingly higher molecular-fragment concentration clearly affected the amide group reorientation dynamics, such that it became increasingly faster and the final Am-I/Am-II ratios for mature SAMs were lower. For example, the ratio asymptotically approached progressively lower values, specifically 0.4, 0.2, 0.2, and 0.1 for the SAMs formed from 100, 50, 20, and 0 mol% of IMHA. The reduction of the surface IMHA concentration accelerated the narrowing of the Am-II band, expressed as the full width at half maximum (FWHM) as well (Figure 4C), which suggests that the increased surface Frag concentration and incubation time synergistically acted on the formation of more homogeneous H-bond strength distribution in the SAM. Overall, the presented data suggest that the relatively bulky imidazole ring group of the IMHA molecule affects the packing ability of the molecules at the amide group and the formation of an extended H-bond network.

**Figure 5:**
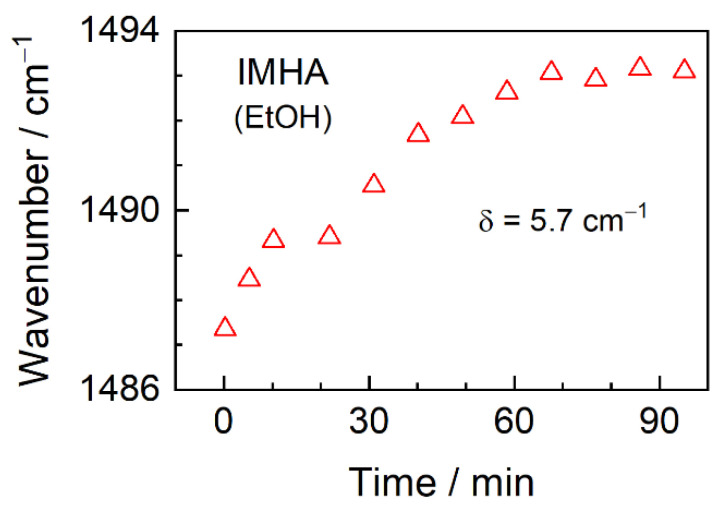
Dependency of the ν(C2–N3) + β(C2H) vibration frequency of the imidazole ring on the SAM formation time.

In our recent study based on the temperature-Raman and theoretical modeling, the Im ring ν(C2–N3) + β(C2H) mode was found to depend on the H-bond strength at the N1/N3 atoms [21]. DFT predicted that interaction of the Im ring with explicit water molecules resulted in a vibrational frequency shift by 2–6 cm^−1^ to higher frequencies, while an addition of two water molecules shifted the vibrational mode by 9 cm^−1^. These data linked higher ν(C2–N3) vibrational frequency with the stronger H-bonding interactions of the Im ring. Here, we observe a wavenumber decrease from 1494 cm^−1^ for bulk IMHA (ATR-FTIR) to 1487 cm^−1^ for IMHA in monolayer (SEIRAS) at initial adsorption times. Such a decrease might be related to reduced H-bonding strength in ethanol-diluted IMHA molecules (0.5 mM) in the incubation solution. As the molecules chaotically adsorbed on a surface, they retained the melted-state character, which, upon reorientation into more concerted and solid-like structures with a gradual SAM development, was lost and resulted in upshifted wavenumbers (Figure 5). The increasing wavenumbers suggest the incrementally stronger H-bond interactions at the Im ring occur at longer incubation times. Notably, the dynamic curve in Figure 5 reaches a plateau in approximately 60 min, which is in contrast to ca. 30 min found for the development of Am-I/Am-II intensities of the IMHA SAM. Thus, the Im ring properties at the interface directly depend on the structure and packing of the interchain amides. 

### 3.3. SEIRAS Analysis of the Structure of IMHA SAMs

**Figure 6:**
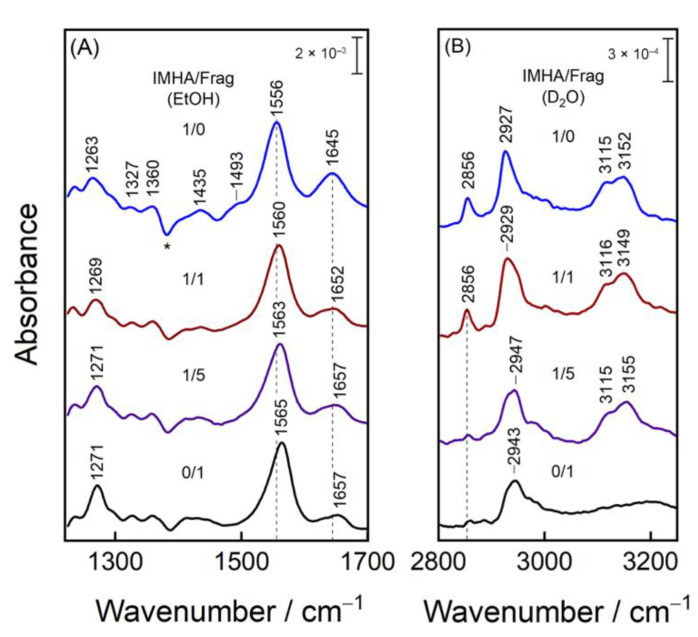
(**A**) SEIRAS spectra of IMHA, binary IMHA/Frag (1/1 and 1/5), and Frag SAMs at the 90th adsorption minute in ethanol incubation solution in 1220–1700 cm^−1^ spectral range. Asterisk marks the negative band, which appears due to the withdrawal of ethanol molecules from the Au surface. (**B**) Spectra of the same composition SAMs after the careful ethanol exchange with D_2_O in the spectral range of 2800–3250 cm^−1^.

To study in detail the way the self-assembled monolayer composition affects its structure, we compared the midrange spectra of various compositions of mature SAMs in ethanol and also the high-frequency spectra of the same SAMs in D_2_O (Figure 6). We performed an ethanol/D_2_O exchange to extract spectral information from the aliphatic and aromatic C–H stretching region, otherwise obscured by strong ethanol-induced aberrations and complex N–H- and O–H-stretching patterns. Analysis of the Am-I/Am-II ratio shows the amide group tendency to become increasingly more perpendicularly oriented with the surface for SAMs, which are composed of higher molecular fragment concentrations (lower concentrations of IMHA), presumably because of reduced sterical interference from the rather bulky neighboring Im ring. Such an assumption is confirmed by an 8.5 cm^−1^ frequency upshift of Am-II mode, which is linked with stronger H-bonding interaction through the N–H group. The decreasing bandwidths from 44 to 41 cm^−1^ for Am-II and from 48 to 35 cm^−1^ for Am-I show that the molecular fragment favors the more uniformly arranged H-bonding network.

The weak mode at 1493 cm^−1^ ascribed to ν(C2–N3) + β(C2H) motion is detected only for the monolayers of the highest IMHA concentrations. Two other Im-related features at 3115 and 3152 cm^−1^ are assigned to ν(C2–H) and ν(C5–H) in Figure 6B. These bands are absent from the molecular-fragment spectrum, confirming the assignment to the =C–H stretching motion of the Im ring. Unexpectedly, the relative intensity of this doublet in the D_2_O environment remains largely unaffected by the IMHA’s concentration in the SAM. This could only be possible if the Im ring group adopts an increasingly more perpendicular surface orientation as its concentration diminishes. Notably, a more perpendicular alignment is observed in D_2_O rather than ethanol-d_6_ solutions (Figure S3), as evidenced by the intensity of the high-frequency C–H stretching doublet. 

In the high-frequency region, 2943–2947 cm^−1^ mode is assigned to the ν_s_(CH_3_) motion of the molecular fragment. The relatively high mode’s position is related to the induction effect produced by the neighboring nitrogen atom, which effectively increases the stretching frequency of the methyl group [33]. The vibrational modes at 2856 and 2927 cm^−1^ are assigned to ν_s_(CH_2_) and ν_as_(CH_2_), respectively. Their intensities depend on the carbon chain alignment with respect to the surface. There is an acute decrease in the intensity of the 2856 cm^−1^ mode when bicomponent 1/1 SAMs is compared to 1/5 (Figure 6B). This could be explained by alkane chains adopting a vertical surface orientation so that the TDMs of their methylene groups are parallel with the surface and no longer SEIRAS-active. We also observe an increased intensity of the molecular-fragment-related symmetric CH_3_ stretching mode. In fact, the absence of its asymmetric counterpart, ν_as_(CH_3_), suggests that methyl groups are being oriented orthogonally with the surface. It is known that the ν_s_(CH_2_) and ν_as_(CH_2_) are highly sensitive to the ordering of the alkane chains so that densely packed and well-ordered SAM absorbs infrared at 2850 and 2917 cm^−1^, whereas those of less-ordered arrangements have absorptions at higher frequencies [43,44,45,46]. Methylene bands are found to be upshifted by ca. 10 cm^−1^ in SEIRAS spectra, suggesting a melted structure of the alkane chains in the SAM, despite the fact that, from the intensity pattern, we found an almost vertical chain arrangement. Obviously, the alkane chain packing is strongly affected by the relatively bulky amide and imidazole ring groups. For comparison, molecules in the bulk phase exhibited wavenumbers nearly the same as the SAM, e.g., 2852 and 2926–2928 cm^−1^. 

We deduce from the SEIRAS spectra of varied-composition SAMs that the decrease in the relative IMHA concentration (i) affected the imidazole ring, amide group, and the alkane chain orientation, prompting them to adopt a more perpendicular orientation to the surface and (ii) strengthened the H-bonding interaction at the N–H group of the amide moiety. These observations imply stiffer head and tail group segments of the mixed SAMs, including an increased flexibility and accessibility to the interactions with the functional imidazole ring group. 

### 3.4. Hydrogen-Bonding Interactions Revealed by Potential-Dependent SEIRAS

**Figure 7:**
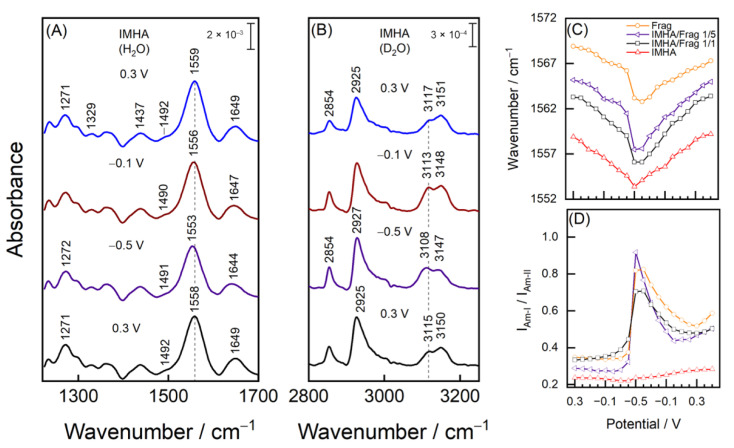
Potential dependent SEIRAS spectra of IMHA monolayer in (**A**) a midrange region in H_2_O and (**B**) C–H stretching region in D_2_O. (**C**) The dependence of Am-II wavenumbers and (**D**) Am-I/Am-II intensity ratio on the electrode polarization.

In order to understand the influence that electric potential has on the structure of mature SAMs and to investigate the H-bonding interactions at Im, series of potential-dependent SEIRAS measurements were performed for various composition SAMs. Figure 7A,B show the spectra of IMHA SAM at electrode polarizations with 0.3, −0.1, and −0.5 V and at 0.3 V after the excursion to negative potentials. Interestingly, both Am-II and Am-I shift to lower wavenumbers by 5–6 cm^−1^ as potential is stepped from 0.3 to −0.5 V, which is related to a decreasing H-bonding strength at N–H site and an increase at the C=O group. We can only speculate possible reasons for such a wavenumber shift discrepancy. One explanation could be that the decreased H-bonding interaction strength between the interchain N–H···O=C groups is related to an increased interaction between the carbonyl and the water molecules (C=O···HOH) at a negatively electrified interface. The Am-II wavenumber dependency on potential is similar for all tested SAM compositions and is related to the gradually weaker H-bonding strength towards the negatively polarized electrode (Figure 7C). The weakening is related to reorientation of the amide planes into a less perpendicular arrangement with the surface (Figure 7D), supported by a sharp Am-I/Am-II ratio increase at –0.5 V for all but the IMHA monolayer. For the IMHA monolayer (Figure 7D red line), strong intermolecular interactions at the Im ring groups bring rigidness to the molecular structure that suppresses amide group flexibility. Other SAM compositions showed a much stronger potential dependency of Am-I/Am-II intensity ratio and an altered Am-I band shape (Figure S4). An unexpected increase in the low-frequency component at 1628–1630 cm^−1^ of the Am-I band was found for each SAM-containing molecular fragment at the −0.5 V bias. Such low wavenumbers in proteins were linked with a rigid and highly ordered β-sheet secondary structure element [30]; however, here we ascribe changes with the reorientation of C=O dipoles more closely along the external field lines (thus the increased Am-I intensity) and the establishment of H-bonding interactions with H_2_O molecules in a liquid solution.

**Figure 8:**
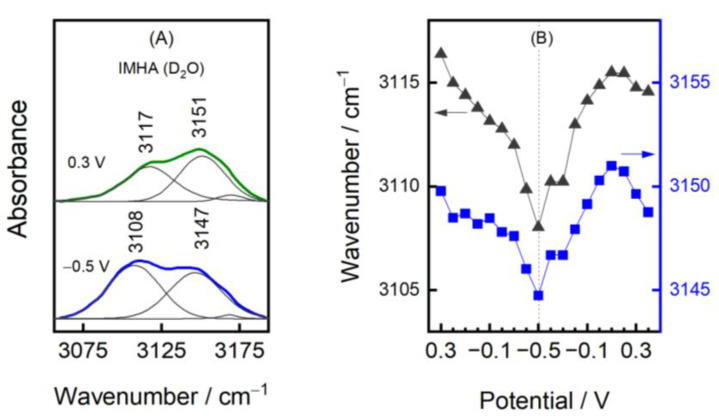
Analysis of the ring C–H stretching modes at 3115 and 3150 cm^−1^. (**A**) SEIRAS spectra of IMHA SAM in D_2_O at 0.3 and –0.5 V potentials in the 3056–3193 cm^−1^ range (colored lines). The experimental spectra are fitted with Gaussian-Lorentzian shape components (grey lines). (**B**) The dependency of 3115 (black triangles) and 3150 cm^−1^ (blue squares) wavenumbers on the electric potential.

The SEIRAS information on the Im ring could be accessed from the 1492 cm^−1^ mode of the monolayer submerged in H_2_O and from a duplet at 3117 and 3151 cm^−1^ in D_2_O. While the former mode is poorly resolved, the duplet intensity suffices to provide a deeper understanding of the biased electric-potential-induced changes in the imidazole ring group. In the case of the T-I form, our DFT calculations and the literature ascribe the lower frequency component at 3117 cm^−1^ to =C2–H stretching and the 3151 cm^−1^ mode to the =C5–H [6,47]. For the molecule in the T-II state, these vibrations become coupled and engage in out-of-phase and in-phase vibrational motions, respectively. Surface adsorption induces the transition from the T-I to the T-II form; therefore, we consider the T-II (N1, N3–D) as the main imidazole tautomeric form in our system [4,21]. The frequency tuning rate of −10.4 and −6.3 cm^−1^V^−1^ for the low and high-frequency modes was identified when electrode polarization was changed from 0.3 to −0.5 V (Figure 8B). Bicomponent monolayers exhibited similar tuning rates of −8.3 and −4.0 cm^−1^V^−1^ for the 1/1 component ratio SAM and −14.1 and −8.5 cm^−1^V^−1^ for the 1/5 ratio SAM.

**Figure 9:**
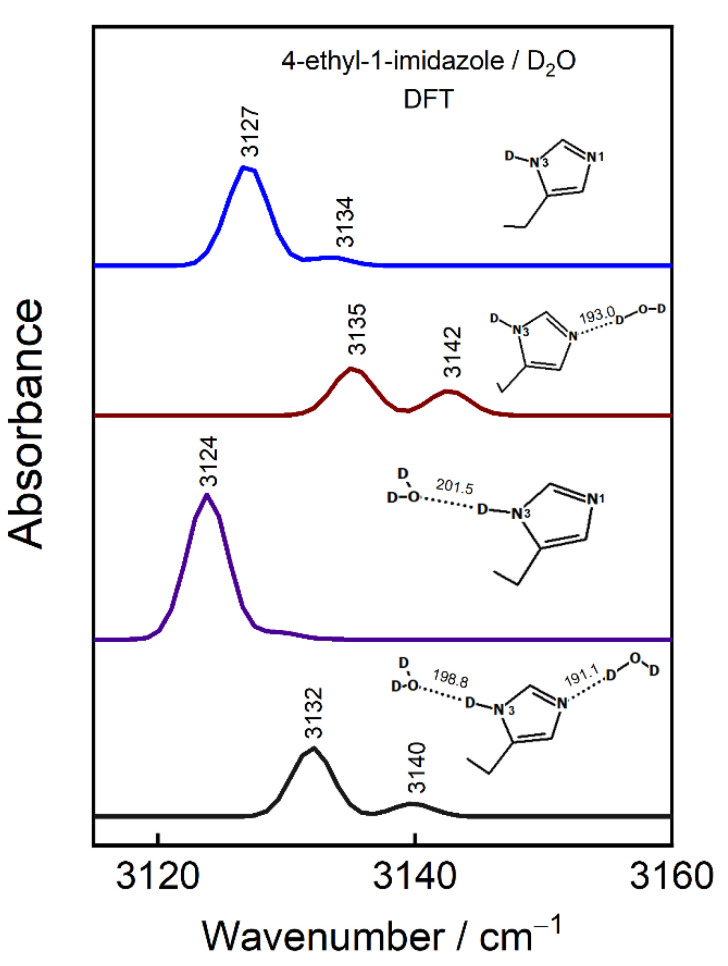
DFT spectra in Im ring =C–H stretching region of 4-ethyl-1-imidazole in T-II form without and with explicit D_2_O molecules at N1 and/or N3–D. Insets show imidazole ring interactions with D_2_O; for detail 3D representation refer to Figure S7. The hydrogen-bonding lengths are indicated in pm. Vibrational frequencies were scaled.

We conducted a DFT modeling of 4-ethyl-1-imidazole in Tautomer-II form (N1, N3–D) with zero, one, and two explicit D_2_O molecules in order to explain the effects that H-bonding interaction has on the vibrational frequency of ν(=C–H) modes (Figure 9). We found that the N1 atom in T-II is more sensitive to hydrogen bonding than the N3–D deuteron. For example, the interaction at N3–D group (N3–D···D_2_O) has little effect on the vibrational frequency of the modes (δ = −3 cm^−1^), whereas the interaction at the N1 atom increases the frequencies by 8 cm^−1^. An almost exact tendency was found for a compound in T-I form. A considerably shorter H-bond at N1 compared to N3–D by 8.5 pm shows the greater stabilization over the ImN1···D_2_O complex. Indeed, a supersonic jet FTIR spectroscopy study suggested that Im prefers accepting the H-bond at N···H−O rather than donating it at N−H···O [48]. However, even stronger Im stabilization was attained with an addition of a second D_2_O molecule to the Im-D_2_O complex. The coordination with two D_2_O molecules resulted in the shortening of both H-bonds by 1.89 and 2.69 pm for the N1 and N3–D sites. The infrared absorption spectroscopic measurements of the model compound imidazole-4-methanol (Im–CH_2_–OH) in solvents of different polarity (Figure S5) clearly shows decreased frequencies of the ν(=C–H) modes in methanol-d_4_, where a less-strong hydrogen-bonding interaction is expected compared to that in D_2_O.

Based on our theoretical and experimental analysis, we linked the =C–H frequency shift towards lower wavenumbers with decreased imidazole H-bond strength. The potential dependency in Figure 8 clearly shows decreasing wavenumbers with negative electrode polarization indicating the reversible reduction of imidazole’s H-bond strength with its immediate surroundings. One can associate such a wavenumber shift with the vibrational Stark effect; however, no significant difference in the frequency tuning rates was found for Im-SAM in the environments of various ionic strengths, as shown in Figure S6. The weakening of the H-bond may be explained by the structural changes of interfacial water. The dielectric constant of water is reduced near the surfaces or under confinement, largely because of the reorientation of water molecule dipoles [49]. The bias electric potential polarizes medium by acting on molecular dipoles and competes against the isotropic H-bond network. Substantially negative polarization orients O–D dipoles to point away from the bulk to the surface [50]. Overall, the arrangement of water molecules at the electrified interface affects the hydrogen interaction strength with exposed Im groups.

The relative intensities of the 3150 and 3115 cm^−1^ modes require some explanation as well. The calculations predict that both displacement vectors of the T-II form of the Im ring lay within the Im plane. The displacement vector of the 3115 cm^−1^ vibrational mode is parallel and points along the C2–H bond direction, whereas the displacement vector of the 3150 cm^−1^ vibrational mode is almost parallel to the N3–D bond direction (Figure S8). Thus, changes in the Im ring surface orientation are reflected in the relative intensities of the 3150 and 3115 cm^−1^ modes. The transition to the negative potentials decreases the I_3150_/I_3115_ intensity ratio, showing that the imidazole tilts towards the position where C2–H is slightly more exposed to the solution. In contrast to the two component SAMs, we found that the potential-induced intensity alterations were slightly dampened for the monolayer composed of 100 mol% IMHA, most likely due to the stronger intermolecular interactions between the Im ring groups. 

## 4. Conclusions

In this work, we studied the imidazole (Im) ring, terminated at alkanethiol molecule self-assembled monolayers (SAMs), formed from IMHA and the surface-dilutor molecular fragment. SAM formation dynamics and interfacial Im ring structure were probed by H/D substitution SEIRAS. We found that despite the SAM composition, the alkane chain-nested amide groups adopt optimal surface orientation within the first 30 min of incubation. Based on the frequency shifting of the Im structure marker band near 1490 cm^−1^, changes in the SAM terminal functional group did not cease until the 60 min of incubation. These changes were linked with the gradual increase of the hydrogen-bonding strength at the Im group. The IMHA SAM formed a structurally rigid organic layer with somewhat disturbed hydrogen-bonding network at amide groups. The introduction of a surface-dilutor molecular fragment affected the Im orientation in such a way that it became more vertically oriented, and the amide groups formed a stronger H-bonding network. Negative electric potentials reversibly decreased the vibrational frequency of Im’s =C–H stretching modes at 3115 and 3150 cm^−1^ (frequency tuning rates were −10.4 and −6.3 cm^−1^V^−1^). We assign a frequency decrease to the weakening of the H-bond interaction of the Im ring with the immediate water at the negatively electrified interface.

## Figures and Tables

**Table 1:** ATR-FTIR, SEIRAS, and DFT vibrational modes and assignments of IMHA and deuterium-substituted IMHA (IMHA-d).

ATR-FTIR		SEIRAS		DFT	Assignement
IMHA	IMHA-d	IMHA	IMHA-d	IMHA-d (T-I)	
Im-related modes					
	3138 vw		3152 m	3158 vw	ν(C5–H)
	3117 vw		3115 m	3139 vw	ν(C2–H)
	1560 vw			1564 vw	ν(C4=C5)
1494 w	1480 sh	1493 vw		1484 w	ν(C2–N3) + β(C2H) + ν(C2–N1) + ν(C5–N1)
Amide-bond-related modes					
1635 s	1622 s	1645 m		1634 s	Am-I or Am-I’, ν(C=O)
1573 s	1467 m	1558 s		1434 m	Am-II, ν(C–N) + δ(NH) orAm-II’, ν(C–N)

Abbreviations: vw, very weak; w, weak; m, medium; s, strong; sh, shoulder; ν, stretching; δ, deformation; β, in-plane; Am-II’, deuterium-substituted Am-II; Am-I’, deuterium-substituted Am-I.

## Data Availability

The data presented in this article are available within this article.

## Supplementary Materials

The following supporting information can be downloaded at: https://www.mdpi.com/article/10.3390/ma15207221/s1, Figure S1: 2D schematic representation of interchain hydrogen-bond network at the amide group of binary IMHA/Frag monolayer on Au. Shaded areas indicate amide group planes, red lines are the approximate directions of Am-I and Am-II transition dipole moment (TDM) vectors; Figure S2: Adsorption time dependency of Am-II wavenumbers of varied composition SAMs; Figure S3: SERAS spectra of IMHA, Frag, and binary (IMHA/Frag 1/1) monolayers in ethanol-d_6_ (EtOD) incubation solution (60 min) and D_2_O; Figure S4: Potential dependent SEIRAS spectra of IMHA, IMHA/Frag 1/1, 1/5, and Frag monolayers in amide region in H_2_O; Figure S5: Infrared absorption spectra in transmission mode of the imidazole-4-methanol (Im–CH_2_–OH) dissolved in methanol-d_4_ and D_2_O; Figure S6: The dependence of ν(=C5–H) and ν(=C2–H) modes positions on the electric potential in the D_2_O solutions of different ionic strengths. SAM was IMHA/Frag 1/1; Figure S7: 3D representation of geometry-optimized 4-ethyl-1-imidazole and D_2_O complexes. The hydrogen bonding lengths are indicated in pm; Figure S8: Displacement vectors calculated for 3115 and 3150 cm^−1^ modes of the 4-ethyl-1-imidazole in Tautomer II form.
