# Supplementary material for: Electrochemical SEIRAS Analysis of Imidazole-Ring-Functionalized Self-Assembled Monolayers
Source: Materials (Basel). 2022 Oct 17;15(20):7221. doi: 10.3390/ma15207221 (PMC9610120; doi:10.3390/ma15207221)
Supplement: Supplementary file 1 [file materials-15-07221-s001.zip › materials-1940142-supplementary.pdf]

SUPPLEMENTARY INFORMATION

for

Electrochemical SEIRAS Analysis of Imidazole-Ring-Functionalized Self-Assembled Monolayers

Vaidas Pudžaitis [1], Martynas Talaikis [2,*], Rita Sadzevičienė [1], Linas Labanauskas [1], Gediminas Niaura [1,2*]

[1] Department of Organic Chemistry, Center for Physical Sciences and Technology (FTMC), Sauletekio Ave. 3, LT-10257 Vilnius, Lithuania

[2] Department of Bioelectrochemistry and Biospectroscopy, Institute of Biochemistry, Life Sciences Center, Vilnius University, Sauletekio Ave. 7, LT-10257 Vilnius, Lithuania

*correspondence: martynas.talaikis@gmc.vu.lt, gediminas.niaura@ftmc.lt

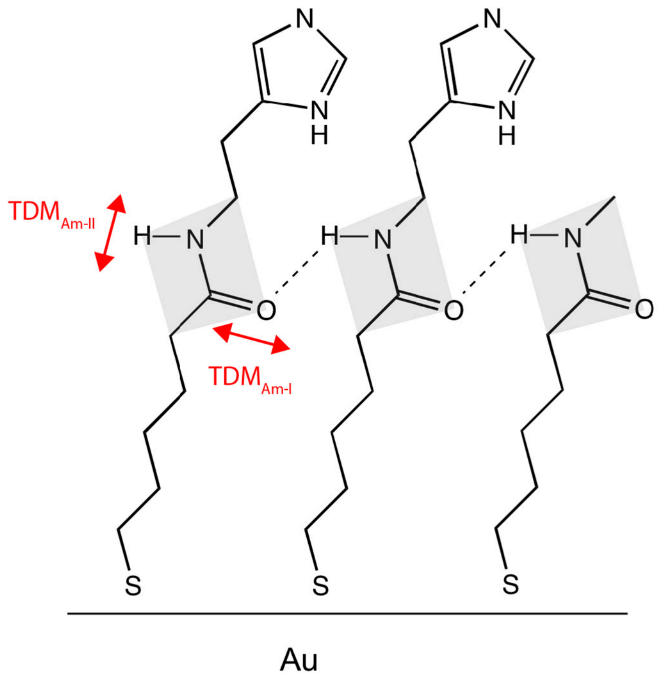

Figure S1. 2D schematic representation of interchain hydrogen bond network at the amide group of binary IMHA/Frag monolayer on Au. Shaded areas indicate amide group planes, red lines are the approximate directions of Am-I and Am-II transition dipole moment (TDM) vectors.

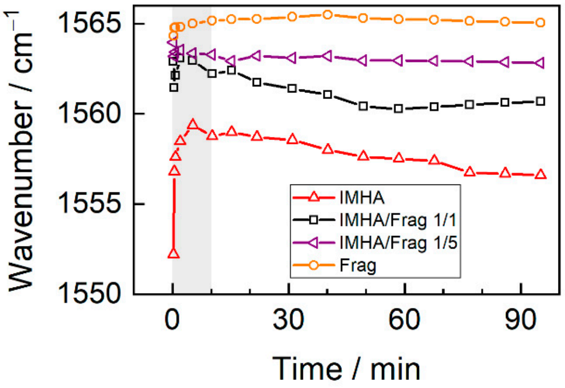

Figure S2. Adsorption time dependency of Am-II wavenumbers of SAMs of varied composition.

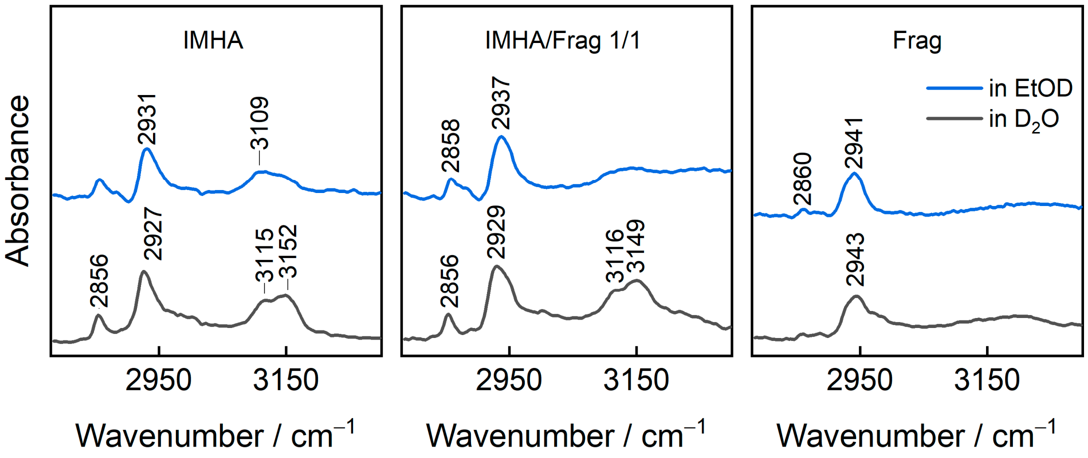

Figure S3. SERAS spectra of IMHA, Frag, and binary (IMHA/Frag 1/1) monolayers in ethanol-d6 (EtOD) incubation solution (60 min) and D_2O.

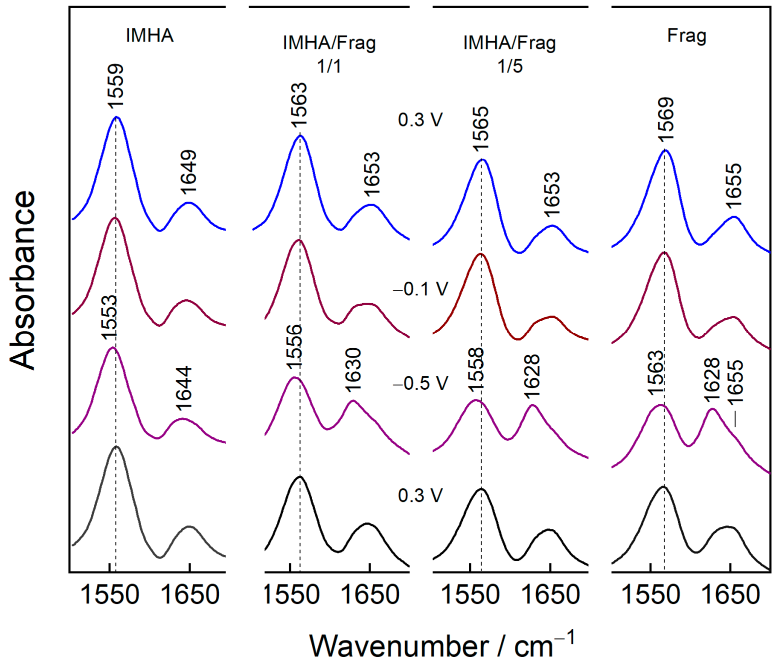

Figure S4. Potential dependent SEIRAS spectra of IMHA, IMHA/Frag 1/1, 1/5, and Frag monolayers in amide region in H_2O.

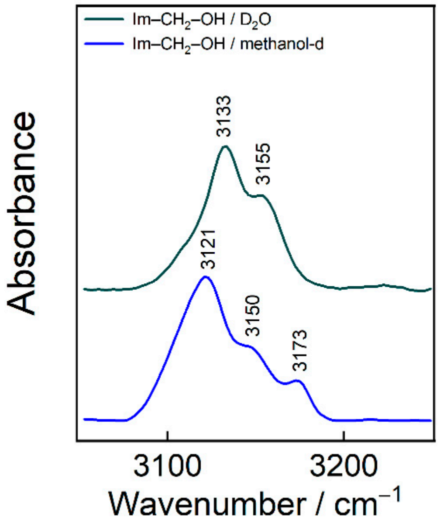

Figure S5. Infrared absorption spectra in transmission mode of the imidazole-4-methanol (Im–CH_2–OH) dissolved in methanol-d4 and D_2O.

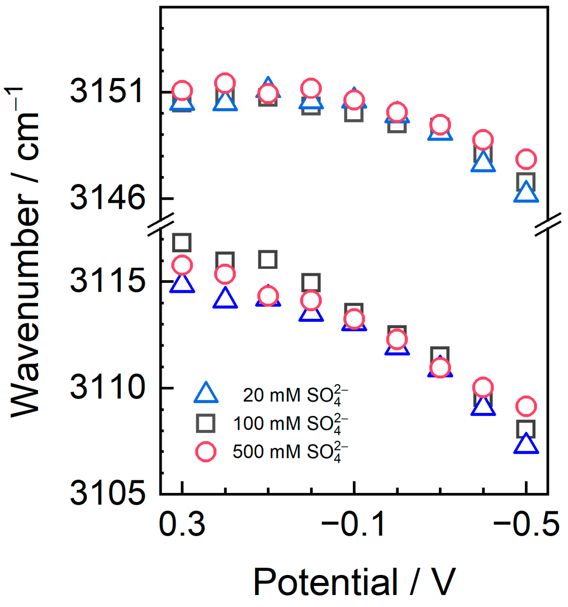

Figure S6. The dependence of ν(=C5–H) and ν(=C2–H) modes positions on the electric potential in the D_2O solutions of different ionic strengths. SAM was IMHA/Frag 1/1.

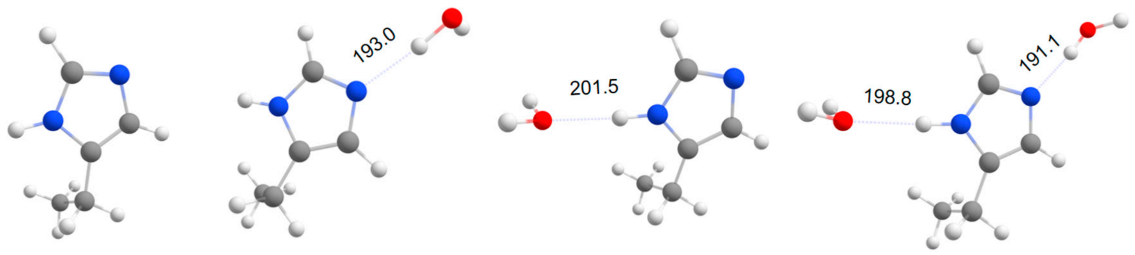

Figure S7. 3D representation of geometry-optimized 4-ethyl-1-imidazole and D_2O complexes. The hydrogen bonding lengths are indicated in pm.

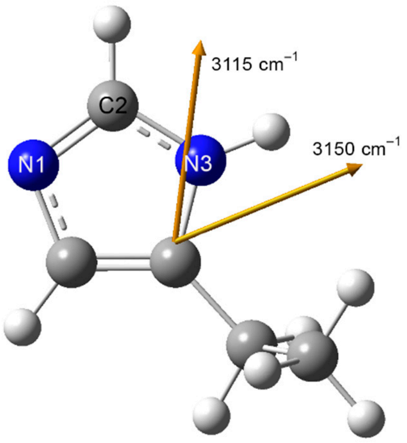

Figure S8. Displacement vectors calculated for 3115 and 3150 cm^{-1} modes of the 4-ethyl-1-imidazole in Tautomer II form.
